# Research Progress on the Mechanism of Ginsenoside Rg1 in Inflammatory Bowel Disease

**DOI:** 10.1155/jimr/9743121

**Published:** 2026-05-28

**Authors:** Dandan Liang, Shaolei Yang, Dehuai Jing, Guangxi Zhou, Fengqin Zhu

**Affiliations:** ^1^ Clinical Medical College of Jining Medical University, Jining Medical University, Jining, Shandong, China, jnmc.edu.cn; ^2^ Department of Gastroenterology, Affiliated Hospital of Jining Medical University, Jining Medical University, Jining, Shandong, China, jnmc.edu.cn

**Keywords:** ginsenoside Rg1, gut microbiota, immune cells, inflammatory bowel disease, intestinal mucosal barrier

## Abstract

Inflammatory bowel disease (IBD), a chronic intestinal inflammatory disorder, has witnessed a rising incidence globally. At present, the primary therapeutic approaches for IBD, such as aminosalicylic acid, glucocorticoids, immunosuppressants, and biologics, often entail notable side effects and limited efficacy. Traditional Chinese medicines (TCMs), particularly ginsenosides, have shown promise in the treatment of IBD. Ginsenoside Rg1 (G‐Rg1), a characteristic constituent of ginsenosides, exerts a therapeutic impact on IBD. This review primarily elaborates on how G‐Rg1 can restore the equilibrium of inflammatory and anti‐inflammatory factors within the intestinal milieu. It achieves this by modifying the structure and metabolism of the intestinal flora, reorganizing the tight junction proteins of the intestinal barrier, and regulating the interplay between signaling pathways and immune cells, ultimately attaining the effect of alleviating IBD.

## 1. Introduction

Inflammatory bowel disease (IBD) represents a group of nonspecific chronic gastrointestinal inflammatory disorders with an unknown etiology. IBD encompasses ulcerative colitis (UC) and Crohn’s disease (CD) [1]. UC is characterized by chronic diffuse inflammation of the colonic mucosa, spreading continuously and symmetrically from the rectum to the proximal segments. Lesions consist of inflammation and ulcers. Primary symptoms are hemorrhagic diarrhea, weight loss, and abdominal cramps, with lesions confined to the colon and rectum. CD can involve all gastrointestinal segments, presenting as chronic granulomatous inflammation and often affecting the terminal ileum and adjacent colon. Most lesions are segmental and asymmetrical, rarely involving the rectum. The main clinical manifestations are abdominal pain, diarrhea, abdominal masses, fistula formation, and perianal lesions, affecting the oral cavity, anus, and entire intestinal tract [2].

Globally, the prevalence of IBD exhibits significant regional and population‐based variations. According to the GIVE‐21 Consortium study published in Nature, currently, most high‐income countries are experiencing a slowdown in the growth of new IBD cases, while the total number of patients continues to increase [3]. At the individual level, the incidence of IBD peaks among individuals aged 50–65, and those with a family history of the disease face a 4–20‐fold higher risk. This condition results from the complex interactions between genetic susceptibility and environmental factors that influence intestinal barrier integrity, immune responses, and microbial balance [4, 5].

The current clinical management of IBD primarily adopts two approaches. The first is nontargeted therapy, which includes 5‐aminosalicylic acid (5‐ASA), immunomodulators, and glucocorticoids. This approach has limitations such as nonspecificity, frequent side effects, hormone dependence/resistance, and substantial long‐term toxicity. The second approach employs targeted therapies using biologics (e.g., infliximab and vedotin) and small‐molecule drugs. However, these treatments present challenges such as immunogenicity, the risk of secondary immune response loss, inconvenient administration, high costs, infection risks, potential systemic side effects that require monitoring, and drug interactions. Notably, current biologics and small‐molecule drugs appear to be effective in only ~30%–40% of patients. Therefore, IBD has been recognized as an intractable disease in modern medicine, and it is of great urgency to develop new medications or adjuvant medications [1, 6, 7].

In recent decades, traditional Chinese medicine (TCM) has demonstrated significant advantages in the research of IBD drugs, characterized by diverse drug sources and few side effects [8]. Currently, dozens of Chinese herbal medicines, including ginsenoside, berberine, curcumin, and baicalin, are utilized for the treatment and alleviation of IBD. Ginsenosides possess various effects, such as hepatoprotection, anti‐inflammation, myocardial protection, antioxidant activity, regulation of intestinal microbial metabolism, neuroprotection, antiangiogenesis, immunosuppression, and renoprotection. Among them, ginsenoside Rg1 (G‐Rg1), as a key active component in ginseng, has shown unique multitarget therapeutic potential in the treatment of IBD. The value of G‐Rg1 lies not only in its broad anti‐inflammatory and immunomodulatory properties but also in the high degree of consistency between these properties and the complex pathogenesis of IBD. Studies have indicated that G‐Rg1 has unique immunomodulator characteristics [9]. Additionally, active metabolites can be generated through unique microbial transformation pathways, and their close interaction with the gut microbiota provides unique advantages for the treatment of IBD, with the specific manifestations of IBD detailed in Tables 1 and 2. It also has a variety of pharmacological activities, such as anti‐inflammation, antioxidant, and antiapoptosis, so this multitarget property makes it an ideal option for the treatment of IBD, a multifactorial disease.

**Table 1:** Primary mechanisms of action of G‐Rg1 in IBD.

Model used	Type of study	Key mechanisms identified	Outcomes	References
DSS‐induced mice	In vivo	Regulating gut microbiota and microbial tryptophan metabolism	Enhanced the state of colonic injury and alleviates colitis symptoms; partially rectified intestinal microbiota dysbiosis; regulated multiple metabolic pathways of intestinal flora, including the valine, leucine, and tryptophan metabolic pathways.	[10]
DSS‐induced mice	In vivo	Regulating the gut microbiota−lipid metabolism−Th1/Th2/Th17 cells axis	Alleviated colitis symptoms in obese mice, ameliorated serum lipid profiles and liver function; modulated the gut microbiota composition in obese mice with colitis; substantially elevated the proportion of helper T cells, and concurrently augmented the ratios of naive T cells and Th2 cells.	[11]
DSS‐induced mice and LPS‐induced RAW 264.7 cells	In vivo and vitro	Regulating Nrf‐2/HO‐1/NF‐κB pathway	Mitigated symptoms related to ulcerative colitis, such as diminishing the extent of weight loss, reducing the DAI score, and augmenting colon length; ameliorated the oxidative stress state; decrease the levels of IL‐1β, IL‐6, and TNF‐α in serum and cell supernatant; elevated the levels of superoxide dismutase in serum, colon, and cell supernatant while reducing malondialdehyde levels; and restored the function of the Nrf‐2/HO‐1/NF‐κB signaling pathway.	[12, 13]
DSS‐induced mice	In vivo	Interfering with the proinflammatory signaling of TLR4‐NLRP12‐NF‐κB	Diminished the secretion of proinflammatory cytokines in dendritic cells through the upregulation of NLRP12 expression；suppressed the release of IL‐1β and TNF‐α, and notably mitigates the inflammatory responses in colitis cytokines levels.	[14]
DSS‐induced mice	In vivo	Regulating memory follicular T cells via Bcl‐6/Blimp‐1 pathway	Enhanced colonic manifestations in obese mice afflicted with ulcerative colitis, mitigated colonic pathological alterations, and diminish the levels of inflammatory cytokines. This is accomplished by augmenting the quantity of central memory follicular helper T cells and reducing the quantity of effector memory Tfh cells, thereby modulating the equilibrium of memory Tfh cell subsets. Concurrently, it downregulates the expression of Bcl‐6 and upregulates the expression of Blimp‐1.	[15]
LPS‐induced mice	In vivo	Regulation of MAPK/NLRP3 signaling pathway	Through the inhibition of the p38 MAPK‐mediated NLRP3 inflammatory signaling pathway and the upregulation of the expression of ZO‐1, occludin, and claudin‐1, the disruption of small intestinal tight junctions induced by LPS was mitigated.	[16]
DSS‐induced mice	In vivo	Engaging in the repair process of the intestinal barrier	Enhanced the pathological features of colonic tissue, alleviate the severity of colitis, concurrently decrease the levels of TNF‐α and FN‐γ in the colon, elevate the levels of IL‐4, and upregulate the expression of ZO‐1 a tight junction protein, in the colon and it repaired the intestinal barrier structure.	[17]
DSS‐induced mice	In vivo	Regulating balanced differentiation of Tfh/Treg cells	Mitigated the inflammatory response in colitis, as evidenced by an increase in body weight and colonic length a decrease in colonic weight, colonic weight index, and histopathological scores, a reduction in the levels of IL‐6 and TNF‐α, and an elevation in the levels of IL‐10. Moreover, it significantly downregulated the levels of PI3K and p‐Akt, and upregulated the expression of PTEN.	[18]
TNBS‐induced mice	In vivo	Inhibiting the binding of LPS to TLR4 on peritoneal macrophages and restoring the equilibrium of Th17/Treg cells	Inhibited TNF‐α and IL‐1β expression in LPS‐stimulated macrophages and suppressed the interaction between LPS‐stimulated macrophages and TLR4. Also impeded colon shortening, myeloperoxidase activity, and the expression of IL‐1β, IL‐17, and TNF‐α induced by TNBS. Moreover, it not only suppressed TNBS‐induced NF‐κB activation but also restored the TNBS‐induced Th17/Treg imbalance and the expression of IL‐10 and Foxp3.	[16]
DSS induced mice	In vivo	Regulating the balance of M1/M2 macrophage polarization and the homeostasis of intestinal flora	Inhibited macrophage activation, regulated the balance of M1/M2 macrophage polarization, and improved the composition of gut microbiota.	[19]

**Table 2:** The major microbial targets of G‐Rg1 in IBD.

Microbes increased/decreased	Type of study	Associated mechanism	Relevance to IBD pathogenesis	References
Increased *norank_f_Muribaculaceae*, *Lactobacillus*, *Allobaculum*, *Akkermansia*, *Sporothrix*, *Bifidobacterium*, *Bacteroides*, *Anaerobic Spirochete;* restrained *Odoribacter*, *Clostridia_UCG-014*, *Bacteroides*, and *Turicibacter*	Animal	Regulating gut microbiota and microbial tryptophan metabolism	Dysbiosis can lead to an increase in the number of potential pathogenic bacteria and a decrease in the proportion of beneficial bacteria. The rise in pathogenic bacteria elevates the risk of intestinal infection and directly induces inflammation, while the reduction in beneficial bacteria results in the loss of anti‐inflammatory and gut homeostasis‐maintaining functions.	[10]
Increased *Rikenellaceae_RC9_gut_group*, *Lachnospiraceae_NK4A136_group*, *Enterorhabdus*, *Desulfovibrio*, and *Alistipes*	Animal	Regulating the gut microbiota–lipid metabolism–Th1/Th2/Th17 cells axis	Dysbiosis disrupts the interaction axis between gut microbiota and immune cells, leading to an imbalance in T‐cell subsets, increased release of proinflammatory cytokines, and reduced anti‐inflammatory factors, thereby exacerbating intestinal inflammatory responses.	[11]

Therefore, G‐Rg1 demonstrates immunomodulatory, intestinal barrier‐protective, and microbiota‐immune axis‐regulating functions, making it a promising candidate for the treatment of IBD. Its multitarget mechanism is in line with the complex pathogenesis of IBD, especially its ability to regulate the intestinal‐specific immune balance, which paves the way for the development of more effective therapeutic strategies. Future research should focus on the clinical validation of G‐Rg1 in IBD patients and its synergistic effects with other anti‐IBD drugs to fully exploit its potential in the comprehensive management of IBD.

## 2. Mechanism of G‐Rg1 on IBD

G‐Rg1, a specific ginsenoside component belonging to the steroid compound category (commonly referred to as triterpene saponins), primarily isolated from ginseng, exhibits potent pharmacological properties and has demonstrated efficacy in alleviating numerous diseases, such as Alzheimer’s disease [20], nonalcoholic fatty liver [21, 22], acute lung injury [23, 24], acute kidney injury [25–27], and so on. The underlying causes and mechanisms of IBD remain elusive. It is hypothesized that IBD results from the interplay of multiple factors, encompassing heredity, infection, psychological state, environment, diet, and local mucosal immune dysregulation [28].

**Figure 1:**
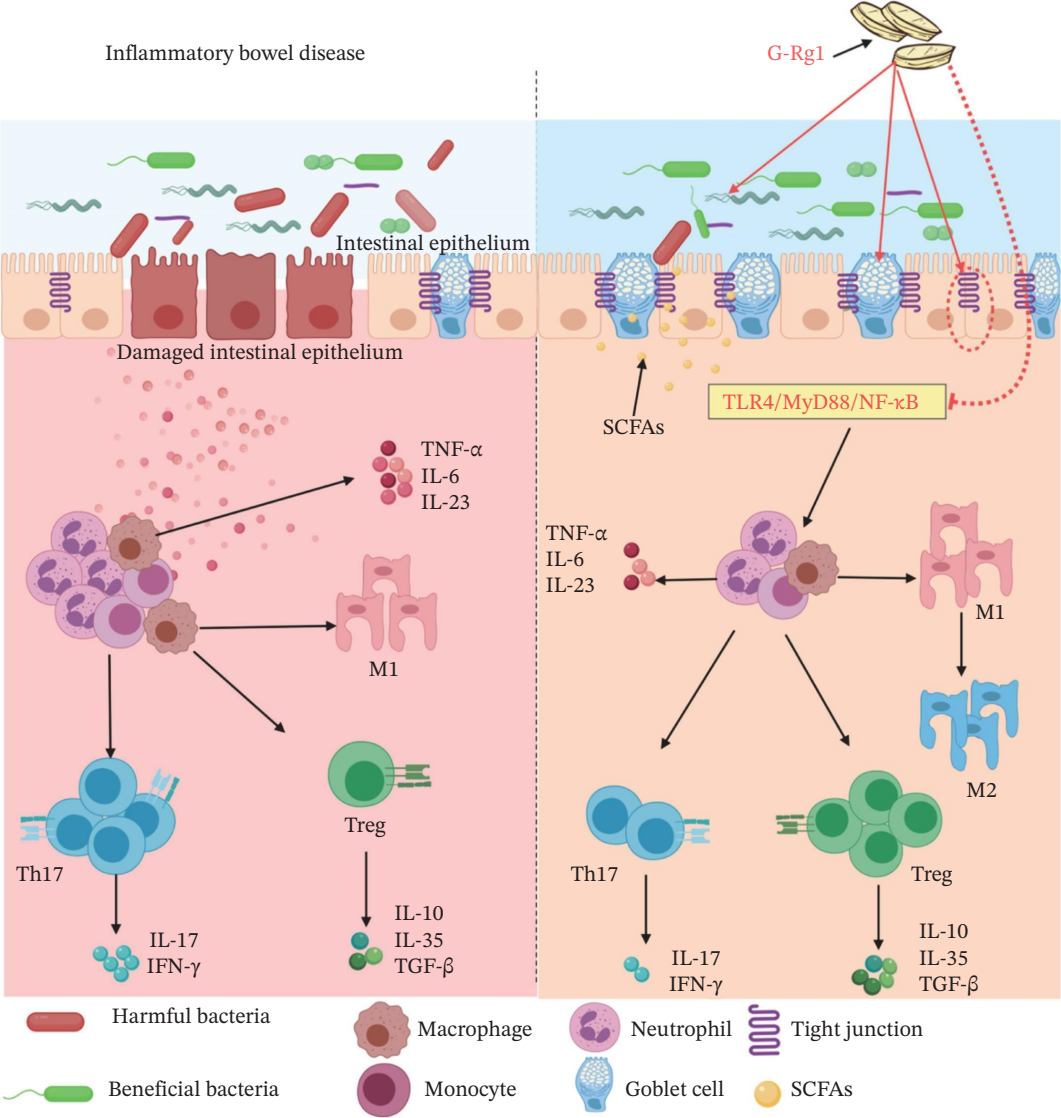
G‐Rg1 has multitarget therapeutic potential for IBD, with its mechanism of action primarily involving three core aspects: regulating the gut microbiota, repairing the intestinal barrier, and modulating immune responses. This substance promotes the proliferation of beneficial bacteria such as *Lactobacilli* and *Bifidobacteria* while inhibiting pathogenic bacteria like clostridia, thereby reshaping the gut microbiota structure. It can directly upregulate the expression of tight junction proteins such as ZO‐1 and claudin‐4, significantly enhancing intestinal barrier function, and promote the secretion of mucus by intestinal epithelial cells, further strengthening intestinal mucosal immune defense. G‐Rg1 induces macrophages to transition from a proinflammatory M1 phenotype to an anti‐inflammatory and reparative M2 phenotype, while regulating the maturation process and antigen‐presenting capacity of dendritic cells, thereby influencing subsequent T‐cell immune responses. It can reshape the balance between Th17 (proinflammatory) and Treg (anti‐inflammatory) cells, modulate the function of follicular helper T cells (Tfh), inhibit the expression of proinflammatory factors (such as IFN‐γ, IL‐17A, and IL‐21), and promote the secretion of anti‐inflammatory factors (such as IL‐4 and IL‐10).

Currently, it is hypothesized that the disruption of intestinal homeostasis may trigger the onset and protract the course of IBD. Under physiological conditions, the maintenance of intestinal homeostasis relies on the equilibrium among intestinal microorganisms, the intestinal epithelial barrier, and the immune system. Therefore, treatment strategies for IBD should take into account microecological reconstruction, restoration of intestinal barrier function, and regulation of the immune system. Consequently, the present study explored the mechanism of G‐Rg1 in inflammatory IBD from three perspectives: microecological reconstruction, intestinal barrier function, and restoration of immune system regulation. The mechanism is presented in Figure 1.

### 2.1. G‐Rg1 Alleviates IBD by Modulating Immune Cells Function

IBD encompasses a combination of innate and adaptive immune responses, which are crucial for maintaining immune equilibrium within the body. A disruption in the immune homeostasis of the intestinal mucosa leads to an excessive inflammatory reaction [29]. Macrophages, neutrophils, and dendritic cells (DCs) serve as the principal innate immune cells within the gut and participate in the pathogenesis of IBD. T cells assume a pivotal role in adaptive immunity throughout the progression of IBD, frequently accompanied by an imbalance of Th17/Treg cells and a reduction in the quantity of negatively regulating regulatory T cells [30, 31].

#### 2.1.1. Macrophages

As a crucial constituent of TCM, G‐Rg1 exerts a therapeutic effect on IBD by modulating macrophages via a multitarget mechanism. Its mechanisms encompass regulating macrophage polarization, enhancing phagocytosis inhibiting inflammatory pathways, and regulating apoptosis and autophagy [32].

In the inflammatory milieu of IBD, macrophages exhibit abnormal polarization towards the proinflammatory M1 phenotype, resulting in the release of a substantial quantity of inflammatory factors [33]. G‐Rg1 effectively suppresses the abnormal activation of the nuclear factor‐kappa B (NF‐κB) signaling pathway, thereby reducing the expression of phosphorylated proteins such as phosphorylated inhibitor of kappa B alpha (p‐IκBα) and phosphorylated NF‐κB. This mechanism facilitates the polarization of macrophages towards the anti‐inflammatory M2 phenotype, thereby restoring the local intestinal immune homeostasis and mitigating tissue injury [19, 34].

Macrophages, regarded as the “scavengers” of the body, play a pivotal role in the clearance of pathogens and cellular debris within the gut. Studies have demonstrated that G‐Rg1 directly enhances the phagocytic function of macrophages. Additionally, G‐Rg1 stimulates the secretion of NO by macrophages, which is essential for immune modulation and pathogen elimination. Collectively, these effects substantially augment the defensive capacity of the intestinal mucosa.

G‐Rg1 exerts anti‐inflammatory and antioxidant effects via multiple pathways. First, it inhibits inflammatory signaling pathways: Apart from suppressing the NF‐κB pathway, G‐Rg1 also inhibits the production of other proinflammatory mediators (e.g., COX‐2) induced by lipopolysaccharide (LPS), thus reducing the secretion of inflammatory factors such as TNF‐α. Second, it activates antioxidant defense mechanisms: G‐Rg1 activates the Nrf2/ARE signaling pathway and upregulates the expression of antioxidant proteins, including HO‐1 and GCLC. This contributes to the reduction of reactive oxygen species (ROS) and superoxide anions, which are key products of oxidative stress, thereby protecting intestinal cells from oxidative damage [35].

Under stressful circumstances, such as serum deprivation, macrophages experience apoptosis. G‐Rg1 upregulates the expression of autophagy‐related proteins, including LC3 and Beclin‐1, thereby inducing protective autophagy. This autophagy promoted by G‐Rg1 effectively inhibits excessive apoptosis in macrophages. When treated with the autophagy inhibitor 3‐methyladenine, the antiapoptotic effects of G‐Rg1 are notably reduced, indicating that its protective function is closely related to autophagy induction. This mechanism is of great significance in maintaining the quantity and functional stability of immune cells within the inflammatory microenvironment of IBD.

In summary, G‐Rg1 exhibits multitargeted therapeutic benefits in the treatment of IBD through the regulation of macrophage polarization, enhancement of their clearance function, suppression of inflammatory cascades and oxidative stress, and the maintenance of a balance between autophagy and apoptosis.

#### 2.1.2. DCs

IBD research has indicated that G‐Rg1 shows potential immunomodulatory significance through the regulation of the DC function [32, 36, 37]. As crucial components in initiating and modulating immune responses, DCs encounter distinct challenges within the intricate intestinal microenvironment of IBD. G‐Rg1 primarily exerts its effects through three mechanisms [14]: (1) precise modulation of DC maturation: G‐Rg1 upregulates the expression of adhesion molecules and costimulatory molecules on the surface of DCs, thereby regulating their maturation process. (2) Preservation of optimal DC maturity: this crucial state enables the activation of appropriate immune responses instead of excessive inflammatory reactions. (3) Facilitation of immune response initiation: through promoting DC maturation, Rg1 effectively contributes to the initiation of appropriate immune responses.

As the most potent specialized antigen‐presenting cells, DCs process and present antigens to T cells, which constitutes the core of adaptive immunity. G‐Rg1 enhances the antigen‐presenting capacity of DCs and stimulates T‐cell proliferation. This enables more efficient elimination of pathogens or abnormal cells and potentially alleviates inflammation in IBD through the regulation of the immune balance [14].

Immune factors function as signaling molecules in intercellular communication. G‐Rg1 promotes the secretion of IL‐12 and the transcription of its mRNA in DCs. IL‐12 plays a crucial role in inducing Th1‐type immune responses and activating natural killer cells, indicating that Rg1 may regulate the immune balance in IBD by modulating the cytokine secretion of DCs [38].

The inflammatory process of IBD is associated with the dysregulation of multiple signaling pathways. Research has indicated that G‐Rg1 can suppress the abnormal activation of signaling pathways like TLR4/NF‐κB/NLRP. Although this description pertains to ginsenosides in general, and the research on the specific signaling pathways of G‐Rg1 DCs is still in progress, it implies that the intervention in critical inflammatory signaling pathways might be a crucial molecular mechanism by which G‐Rg1 modulates DC functions and alleviates IBD [39].

In conclusion, G‐Rg1 modulates the dysregulated immune response in IBD by promoting the maturation of DCs, enhancing antigen presentation and cytokine secretion, and regulating crucial inflammatory signaling pathways. Consequently, G‐Rg1 emerges as a promising candidate for immunomodulatory therapy in IBD.

#### 2.1.3. Neutrophils

During the progression of IBD, the abnormal activation and infiltration of neutrophils emerge as crucial factors contributing to intestinal tissue damage [40]. Research findings suggest that G‐Rg1 can modulate neutrophil function via multiple pathways, thereby exerting protective effects in the treatment of IBD. Neutrophils, as the first immune cells to arrive at inflammatory loci, display excessive activation and dysfunction, which exacerbates intestinal damage in IBD. G‐Rg1 mainly exerts its effects through the following mechanisms: inhibition of abnormal adhesion and migration: in the inflammatory milieu of IBD, activated neutrophils interact with platelets, adhere to the vascular endothelium, and migrate to intestinal tissues, releasing detrimental substances. G‐Rg1 significantly inhibits thrombin‐activated platelet–neutrophil adhesion. This inhibitory action aids in reducing the abnormal aggregation and infiltration of neutrophils in intestinal inflammation, potentially mitigating their destructive influence on intestinal tissues. Moreover, G‐Rg1 exhibits thrombosis‐prolonging properties, which may have a positive impact on ameliorating the intestinal microcirculation disorders associated with IBD [41].

Timely apoptosis plays a crucial role in the normal resolution of neutrophil‐mediated inflammatory responses. G‐Rg1 significantly inhibits neutrophil apoptosis through the activation of the PI3K/AKT and ERK1/2 signaling pathways. This antiapoptotic effect extends neutrophil survival, theoretically augmenting their capacity to eliminate pathogens and necrotic debris at inflammatory loci. Nevertheless, within the complex milieu of IBD, whether this effect is unequivocally beneficial necessitates assessment within the context of the overall inflammatory state [42, 43].

Phagocytosis represents the core mechanism by which neutrophils eliminate invading pathogens. Research indicates that G‐Rg1 significantly elevates neutrophil phagocytosis rates and indices. This implies that G‐Rg1 enhances neutrophils’ ability to recognize, engulf, and eliminate noxious substances such as bacteria, thereby improving the control of intestinal infections and maintaining the stability of the microbial community [42].

#### 2.1.4. Th Cells

The regulatory effects of G‐Rg1 on T cells in IBD mainly originate from its multitargeted intervention in T‐cell activation, differentiation, and function, thus restoring the impaired intestinal immune homeostasis. In the intricate IBD milieu, T‐cell dysfunction acts as the core mechanism beneath intestinal immune imbalance and chronic inflammation [44]. G‐Rg1 exerts precise regulation through the following mechanisms:

Follicular helper T cells (Tfh) assume a crucial role in facilitating B cells to generate antibodies and regulating immune responses. In experimental colitis models, it has been demonstrated that G‐Rg1 can preserve the homeostasis of Tfh cell subsets. It not only stimulates the secretion of anti‐inflammatory factors IL‐4 and IL‐10 but also effectively inhibits the expression of proinflammatory factors, including interferon‐γ (IFN‐γ), IL‐17A, and IL‐21. This regulation of Tfh cells represents a key mechanism through which Rg1 ameliorates the symptoms of colitis [15, 45].

In IBD, the equilibrium between proinflammatory T helper 17 (Th17) cells and anti‐inflammatory Treg is disrupted. Research findings suggest that G‐Rg1 can modulate the differentiation along the Th17/Treg axis through the regulation of the gut microbiota. By restoring the proportional equilibrium of these cell types, G‐Rg1 contributes to the reestablishment of intestinal immune tolerance, thus addressing the fundamental cause of abnormal autoimmune attacks [16, 18].

The normal functioning of T cells is contingent upon their underlying signaling pathways. Molecular docking analysis reveals that Rg1 can effectively bind to targets within the TLR/MyD88 pathway, such as TLR2 and MyD88. By obstructing this crucial pathway, G‐Rg1 impedes the upstream signaling that induces excessive T‐cell activation, consequently mitigating downstream inflammatory cascades [15, 46].

In addition to complex immune network regulation, G‐Rg1 also demonstrates direct effects. Studies have found that G‐Rg1 can significantly inhibit T lymphocyte proliferation induced by jack bean protein A (Con A). This indicates that under specific conditions, G‐Rg1 can directly suppress excessive T‐cell immune responses.

#### 2.1.5. Others Immune Cell

Beyond neutrophils, macrophages, DCs, and T cells, G‐Rg1 can also exert effects on other cell types [47]. B lymphocytes play a complex role in IBD. They contribute to the maintenance of intestinal mucosal homeostasis through the secretion of immunoglobulin A (IgA), yet they may also exacerbate inflammation by producing pathogenic IgG [48, 49]. G‐Rg1 may influence B‐cell differentiation and antibody synthesis via its extensive immunomodulatory functions. For example, it may contribute to restoring the imbalance between the protective IgA2 and proinflammatory IgG, which is commonly observed in IBD. Moreover, G‐Rg1 might facilitate the expansion of regulatory B cell subsets with negative immunomodulatory functions, augmenting their production of IL‐10 to indirectly suppress excessive inflammatory responses.

As essential innate immune cells, NK cells exhibit altered activation patterns and functional states in IBD [50, 51]. Research indicates that NK cells from IBD patients present distinct receptor expression profiles (e.g., killer‐cell Ig‐like receptor (KIR) DS subtypes) compared to those from healthy individuals. This may enhance their cytotoxicity and contribute to the disease progression. G‐Rg1 could regulate NK cell cytotoxicity and IFN‐γ secretion by balancing activating and inhibitory receptors or modulating the cytokine microenvironment of key mediators such as IL‐12, IL‐15, and IL‐18, thereby maintaining the equilibrium between abnormal cell clearance and immune tolerance [30].

In conclusion, although the direct mechanisms of G‐Rg1’s effects on B cells, NK cells, and NKT cells in IBD necessitate further investigation [52], its well‐established multitarget and bidirectional immunomodulatory properties imply that it may similarly regulate these immune cells.

### 2.2. G‐Rg1 Improves IBD Through Intestinal Mucosal Barrier Repair

The intestinal mucosa serves as the most extensive barrier within the human body, exhibiting a defensive function against microorganisms and food antigens [53]. The outermost component of this barrier is the mucus layer, primarily constituted by mucins, antimicrobial peptides, and secretory Ig A (sIgA), which represents the initial point of interaction with the gut microbiota. Research has indicated that in patients with IBD, there is an upregulation of claudin‐2, a downregulation of occludin, and activation of epithelial myosin light‐chain kinase in the intestinal mucosa, resulting in the disruption of the intestinal mucosa. Moreover, barrier repair has emerged as a novel therapeutic target for IBD.

Research has demonstrated that G‐Rg1 can upregulate tight junction proteins such as zonula occludens‐1 (ZO‐1) and claudin‐4, thereby enhancing intestinal barrier function significantly for addressing the characteristic increase in intestinal permeability in IBD. Additionally, G‐Rg1 promotes intestinal sIgA secretion, further strengthening mucosal immune defenses [17].

Studies have also suggested that certain factors related to gut homeostasis (such as Brahma‐related gene 1 mentioned in the research) can influence intestinal homeostasis by regulating the transcription of autophagy‐related genes (e.g., autophagy‐related 16 like 1, activating molecule in Beclin1‐regulated autophagy protein 1, and autophagy‐related 7). Maintaining normal autophagy function is essential for preventing apoptosis in intestinal epithelial cells and preserving the barrier integrity. Although this study specifically focuses on BRG1 rather than G‐Rg1, it reveals the crucial role of autophagy in the development of colitis and tumors, providing a theoretical basis for understanding how G‐Rg1 may similarly impact intestinal health through analogous pathways [54–56].

### 2.3. G‐Rg1 Alleviates IBD by Adjusting the Composition and Metabolism of Gut Microbiota

The gastrointestinal tract is regarded as the “second‐largest organ” in the human body and assumes a pivotal role in sustaining physical well‐being. Nevertheless, individuals suffering from IBD frequently experience dysbiosis of the gut microbiota, leading to a reduction in the beneficial gut microbiota and an elevation in pathogenic bacteria such as *Escherichia coli*, *Enterococcus faecalis*, and *Fusobacterium nucleatum*. Moreover, metabolites of the gut microbiota, encompassing trptophan, short‐chain fatty acids (SCFAs), and bile acids, can impact the advancement of IBD [57].

#### 2.3.1. The Influence of Rg1 on Gut Microbiota Composition

The regulation of gut microbiota by G‐Rg1 plays a pivotal role in its multitargeted mechanism for the treatment of IBD. A substantial body of research has demonstrated that G‐Rg1 is not merely passively metabolized by the microbial community; rather, it actively and selectively regulates the equilibrium of the intestinal flora, thereby exerting indirect anti‐inflammatory and immunomodulatory effects.

G‐Rg1 notably alleviates intestinal dysbiosis by effectively increasing the relative abundance of beneficial bacterial taxa, including *Lactobacillus* and *Bifidobacterium*, and significantly reducing the colonization of opportunistic pathogenic bacteria, such as *E. coli* and *Enterococcus*. Moreover, G‐Rg1 has positive impacts on the overall diversity of the gut microbiota, as shown by the elevated values of α‐diversity indices (e.g., Shannon index and Chao1 index) and the optimized β‐diversity structure, thus facilitating the overall equilibrium and well‐being of the intestinal microecosystem [10].

G‐Rg1 has been shown to specifically increase the relative abundance of beneficial bacteria, such as *Lactobacillus* and *Bifidobacterium*, which generate SCFAs and maintain the function of the intestinal barrier. Simultaneously, G‐Rg1 effectively suppresses the proliferation of opportunistic bacteria associated with intestinal inflammation, including certain Gram‐negative bacteria. This optimized microbiota structure contributes to the reduction of the production of proinflammatory factors, such as LPS, in the gut, thus alleviating the abnormal activation of the immune system at its origin [11].

#### 2.3.2. The Influence of Rg1 on Gut Microbiota Metabolism

Recent advances in metabolomics have revealed the important role of intestinal flora metabolism. The intestinal flora provide nutrients to the host and regulate intestinal immunity through various metabolic pathways. They metabolize cellulose, tryptophan, arginine, and other substances in food to produce SCFAs, indoles, and polyamines, which affect intestinal immunity by acting on intestinal epithelial cells [58].

Additionally, the intestinal flora metabolize the primary bile acids secreted by the body to generate secondary bile acids, taurine, and other substances that participate in immunity by acting on innate immune cells such as DCs and macrophages.

In patients with IBD, a notable disruption of the intestinal metabolome can be detected, which is manifested by abnormalities in the metabolism of SCFAs, bile acids, and tryptophan, among other substances [11].

For instance, a multitude of studies have indicated that the abundance of SCFA‐producing beneficial gut bacteria (such as *Faecalibacterium prausnitzii* and *Roseburia*) in the intestinal microbiota of IBD patients is diminished, leading to significantly reduced levels of SCFAs. This deficiency undermines the anti‐inflammatory and barrier repair functions of SCFAs and serves as a crucial factor in driving the progression of IBD.

During the inflammatory process of IBD, an impaired intestinal barrier and an activated immune response establish a complex microenvironment. Research has revealed that the mechanism of G‐Rg1 not only involves reducing the level of SCFAs but also rectifying the disorder of SCFA metabolism and restoring intestinal homeostasis through multitarget actions. G‐Rg1 exerts anti‐inflammatory and barrier‐protective effects by augmenting SCFA levels. G‐Rg1 has long‐term effects on promoting the proliferation of beneficial bacteria and restoring the physiological levels of SCFAs. Consequently, the vicious cycle of dysbiosis–SCFAs deficiency–barrier damage–immune activation can be disrupted, and the homeostasis of the intestinal microenvironment can be reestablished.

Several investigations have proposed that G‐Rg1 has the potential to regulate the gut microbiota. If G‐Rg1 promotes the growth of beneficial bacteria such as *Clostridium*, which produce indole compounds such as indole propionic acid (IPA), it may indirectly enhance intestinal barrier function and local immune homeostasis. This is directly related to the gut microbiota pathway in tryptophan metabolism as various tryptophan metabolites require binding to receptors, such as AhR, to exert their functions. G‐Rg1 may intervene in these downstream signaling pathways by enhancing AhR signaling, thereby amplifying the anti‐inflammatory and barrier repair effects of beneficial metabolites [10].

Patients with IBD frequently display bile acid metabolic disorders, characterized by decreased bile salt hydrolase activity and an imbalance between primary and secondary bile acids. Secondary bile acids such as lithocholic acid (LCA) and deoxycholic acid (DCA) exert immunomodulatory effects by activating TGR5 receptors and farnesyl X receptors (FXR) [53]. G‐Rg1 may restore bile acid metabolic balance by regulating the gut microbiota and influencing bile acid conversion [59–63].

In conclusion, G‐Rg1 may play a regulatory role in the intestinal metabolic disorders of IBD through multiple mechanisms including the modulation of gut microbiota metabolites (SCFAs, bile acids, and tryptophan metabolites), intervention in immune signaling pathways (TLR/MyD88), and enhancement of intestinal barrier function.

## 3. Conclusion

IBD is a chronic autoimmune intestinal inflammatory disorder with an undetermined etiology involving environmental, infectious, immune, and genetic factors. The primary clinical manifestations encompass diarrhea, abdominal pain, and purulent mucous stools.

G‐Rg1, a ginsenoside extract, possesses multiple pharmacological attributes, including microecological regulation, immunomodulation, and anti‐inflammatory effects. It assumes a pivotal role in regulating the intestinal flora, reconstructing tight junctions, and modulating the immune response in IBD.

G‐Rg1 can modulate the composition of the gut microbiome. It elevates the abundance of certain beneficial bacteria (such as *Bacteroidetes*) and reduces the abundance of harmful bacteria (such as *Proteus*). This modulation may be accomplished by influencing the microecological milieu in the gut, facilitating the growth and reproduction of beneficial bacteria, and inhibiting the activity of harmful bacteria.

The metabolic products generated by the gut microbiome exert significant impacts on the physiological and pathological states of the host. G‐Rg1 may regulate the metabolic activity of the gut microbiome to alter the types and quantities of metabolic products in the gut, thereby affecting the host’s immune and inflammatory responses. For instance, G‐Rg1 may promote the production of anti‐inflammatory SCFA by beneficial bacteria or inhibit the production of proinflammatory factors by harmful bacteria.

The integrity of the intestinal barrier is of paramount importance for preventing the ingress of harmful substances into the body. G‐Rg1 may regulate the composition and metabolic activity of the gut microbiome to enhance the integrity of the intestinal barrier, diminish intestinal permeability, and prevent the entry of harmful bacteria, toxins, and other substances into the bloodstream, thus mitigating systemic inflammatory responses.

The gut microbiome interacts closely with the host immune system. G‐Rg1 may regulate the composition and metabolic activity of the gut microbiome to influence the differentiation and function of host immune cells, thereby modulating the host’s immune response. For example, G‐Rg1 may promote the differentiation of Treg and inhibit the production of proinflammatory cytokines, thereby alleviating intestinal inflammation.

While the aforementioned research findings are promising, substantial challenges persist regarding the application of G‐Rg1 in IBD treatment. Current studies on G‐Rg1 therapy for IBD predominantly rely on DSS‐ or TNBS‐induced colitis mouse models, which fail to reflect the complex pathological processes and heterogeneity of human IBD. Our understanding of G‐Rg1’s absorption, distribution, metabolism, and excretion in vivo remains inadequate. Although the multitarget characteristics of G‐Rg1 confer advantages, they also complicate the identification of its key mechanisms.

To promote the clinical translation of Rg1, future research should concentrate on the following aspects: utilizing advanced technologies such as cell‐specific gene knockout animals to further validate the critical targets of Rg1; investigating the effects of Rg1 on other immune cells (e.g., B cells and innate lymphocytes) and apoptosis pathways (e.g., pyroptosis and ferroptosis) in IBD to comprehensively delineate its functional network; conducting high‐quality preclinical safety and efficacy evaluations, followed by rigorous clinical trials to validate the therapeutic efficacy and safety of Rg1 in IBD patients. Given the complexity of IBD treatment, exploring the synergistic effects between Rg1 and existing standard therapies (e.g., mesalazine) may offer novel therapeutic strategies.

In conclusion, this study demonstrates that G‐Rg1 exhibits promising therapeutic potential in experimental colitis through multiple mechanisms, including immune regulation, anti‐inflammatory effects, intestinal barrier protection, and gut microbiota modulation. Currently, Rg1 research is in the preclinical stage, facing challenges like model limitations and insufficient pharmacokinetic understanding. Most studies use animal or cell models, different from human disease pathophysiology, especially in neurodegenerative disorders. The multitarget and multipathway features of Rg1 have pros and cons. Its core targets and main mechanisms are unclear, making clinical efficacy and risk assessment difficult. Preclinical broad‐spectrum efficacy may be reduced or cause unintended interactions in humans. For pharmacokinetics, Phase I studies in healthy volunteers are needed to clarify human absorption, distribution, metabolism, and excretion, focusing on low oral bioavailability and gut microbiota‐mediated metabolism. In terms of safety, though Rg1 is traditionally considered safe, a systematic evaluation of long‐term high‐dose administration to humans is crucial, concentrating on its hormone‐like activity’s effects on the endocrine system and drug interactions. Based on pharmacokinetic and safety data, a dosage range should be set for Phase II trials, which can explore nonlinear dose–response relationships and solve issues like low oral bioavailability and poor blood–brain barrier penetration. Considering Rg1’s long‐term use, preliminary safety data, and preclinical efficacy evidence, one to two indications can be chosen for proof‐of‐concept studies. However, these studies require high‐purity Rg1 raw materials with well‐defined components and controllable quality, compliance with GMP production standards, and preclinical pharmacological and safety assessments of candidate formulations for clinical translation.

## Author Contributions

Dandan Liang conceived the study, wrote the original manuscript, and designed the figure. Shaolei Yang and Dehuai Jing contributed to interpreting the figure, reviewing and revising the manuscript critically for intellectual content. Guangxi Zhou and Fengqin Zhu conceived the idea and designed the manuscript.

## Funding

This work was supported by the National Natural Science Foundation of China (Grants 82270562 and 82200591), the National Natural Science Foundation of Shandong Province (Grants ZR2025MS1260 and ZR2024MH282), the Development Plan of Medicine and Health Science and Technology in Shandong Province (Grant 202503030856), and the TCM Science and Technology Development Plan of Shandong Province (Grant M20254301).

## Disclosure

All authors gave final approval for the submission and agree to be accountable for all aspects of the work.

## Ethics Statement

The authors have nothing to report.

## Conflicts of Interest

The authors declare no conflicts of interest.

## Data Availability

Data sharing is not applicable to this article as no new data were created or analyzed in this study.
